# Response gene to complement 32 regulates the G2/M phase checkpoint during renal tubular epithelial cell repair

**DOI:** 10.1186/s11658-016-0021-1

**Published:** 2016-09-20

**Authors:** Yun-lin Shen, Hua-jie Liu, Lei Sun, Xiao-ling Niu, Xin-yu Kuang, Ping Wang, Sheng Hao, Wen-yan Huang

**Affiliations:** Department of Nephrology and Rheumatology, Shanghai Children’s Hospital, Shanghai Jiao Tong University, Shanghai, 200062 China

**Keywords:** Response gene to complement 32, Cell cycle, G2/M phase, Tumor necrosis factor-alpha, Tubulointerstitial fibrosis, Tubular epithelial cell repair

## Abstract

**Background:**

The aim of this study was to evaluate the influence of RGC-32 (response gene to complement 32) on cell cycle progression in renal tubular epithelial cell injury.

**Methods:**

NRK-52E cells with overexpressed or silenced RGC-32 were constructed via transient transfection with RGC-32 expression plasmid and RGC-32 siRNA plasmid, and the cell cycle distribution was determined. The expression levels of fibrosis factors, including smooth muscle action (α-SMA), fibronectin (FN) and E-cadherin, were assessed in cells with silenced RGC-32.

**Results:**

The cells were injured via TNF-α treatment, and the injury was detectable by the enhanced expression of neutrophil gelatinase-associated lipocalin (NGAL). RGC-32 expression also increased significantly. The number of cells at G2/M phase increased dramatically in RGC-32 silenced cells, indicating that RGC-32 silencing induced G2/M arrest. In addition, after treatment with TNF-α, the NRK-52E cells with silenced RGC-32 showed significantly increased expression of α-SMA and FN, but decreased expression of E-cadherin.

**Conclusions:**

The results of this study suggest that RGC-32 probably has an important impact on the repair process of renal tubular epithelial cells in vitro by regulating the G2/M phase checkpoint, cell fibrosis and cell adhesion. However, the exact mechanism needs to be further elucidated.

## Background

Available data suggest that acute and chronic kidney injury have become global health problems [1, 2]. After injury, the kidney has intrinsic repair capability through its surviving tubular epithelial cells [3]. Renal tubular epithelial tissue plays a vital role in the processes of post-injury germination and development, and in the prognosis of kidney injury [4–6]. The mechanisms of renal tubular injury and repair are known to be rather complex processes, involving cell cycle regulation, the signal transduction pathway and cell behavior changes. However, there is a lack of detailed studies on these mechanisms.

Our previous study found that response gene to complement 32 (RGC-32), also known as regulator of cell cycle (RGCC), is critical for renal tubulointerstitial fibrosis and plays an important role in epithelial–mesenchymal transition (EMT) [7]. Simultaneously, RGC-32 is considered a key factor in cell cycle regulation [8–12].

RGC-32 is induced by p53 in response to DNA damage or by sublytic levels of complement system proteins [9]. It is expressed and involved in cell cycle activation in the endothelial cells of the kidney, pancreas, liver and some other organs [12]. Studies have shown that RGC-32 is essential for fibroblast activation in renal fibrosis [13, 14]. However, the role of RGC-32 in the regulation of the cell cycle during renal tubular epithelial cell repair remains unclear.

This study was carried out to evaluate the influence of RGC-32 on the cell cycle during renal tubular epithelial cell repair after acute injury, which was induced with tumor necrosis factor-alpha (TNF-α). NRK-52E cells with overexpressed and silenced RGC-32 were designed via transient transfection to explore the influence of RGC-32 on the cell cycle. Finally, the cells with silenced RGC-32 were treated with TNF-α to investigate the changes in fibrosis factors. We anticipate that our findings will provide a basis for the treatment of renal tubular epithelial cell injury.

## Methods

### Cell culture

NRK-52E cells (the normal rat kidney cell line CRL-1571) were purchased from the American Type Culture Collection. Cells were cultured as described previously [15]. Briefly, NRK-52E cells were cultured in Dulbecco’s modified Eagle’s medium (DMEM; GIBCO) with 5 % fetal bovine serum and 4 mM L-glutamine at 37 °C in a 95 % air and 5 % CO_2_ incubator.

### Construction of RGC-32 expression plasmid and short hairpin interfering RNA

The RGC-32 expression plasmid was constructed as previously described [16]. Briefly, RGC-32 cDNA was amplified from mRNA extracted from TGF-β-treated NRK-52E cells. The 5′ sense primers included a BamHI restriction site for cloning, a Kozak sequence and a T7 tag followed by an RGC-32 cDNA sequence. The 3′ primer included the RGC-32 cDNA sequence, a stop codon and an XbaI restriction site. RGC-32 full-length cDNA was amplified with Vent DNA polymerase (New England Biolabs). The amplification product and pcDNA 3.0 vector were digested with BamHI and XbaI and then purified, followed by ligation with T4 DNA ligase (New England Biolabs). The specificity of the resulting clone was verified via sequencing. RGC-32 overexpression in NRK-52E cells was confirmed via western blot using anti-T7 antibody (Novagen).

The RGC-32 shRNA plasmid was constructed as described previously [7]. Double-stranded DNA oligonucleotides for RGC-32 and scrambled (control) shRNA were designed using siRNA Target Designer (Promega). The RGC-32 shRNA sequence was CGGCCATTCTTGGTTCACTATTCAAGAGATAGTGAACCAAGAATGGCCCT and the scrambled shRNA sequence was CGCCTCTCTCTTAGTGAGATTTCAAGAGAATCTCACTAAGAGAGAGGCCT. shRNA DNA templates were inserted into pGeneClip vectors using GeneClip U1 hairpin cloning systems (Promega) following the manufacturer’s recommendations. The sizes and sequences of inserts were verified via sequencing.

### Transient transfection

NRK-52E cells were transfected with RGC-32 expression plasmid and RGC-32 shRNA plasmid according to previously reported procedures [6, 7]. Briefly, NRK-52E cells were plated at 3 × 10^5^ cells/well in 6-well plates and incubated until they reached 80 % confluence. Cells were then transiently transfected in triplicate with Lipofectamine 2000 (Invitrogen) according to the manufacturer’s recommendations.

### TNF-α-induced acute injury to NRK-52E cells

TNF-α is a cell signaling protein involved in systemic inflammation. It is one of the cytokines present in the acute phase reaction. NRK-52E cells were seeded in 6-well plates with 3 × 10^5^ cells/well and incubated until they reached 80 % confluence. The cells were starved in serum-free DMEM for 6 h, and then treated with 10 ng/ml TNF-α for 0, 6, 12 and 24 h. The injury was evaluated by determining the protein expression level of neutrophil gelatinase-associated lipocalin (NGAL), a biomarker of kidney injury [17], via western blotting.

### Western blot analysis

NRK-52E cells were washed twice with PBS, followed by protein extraction using RIPA buffer consisting of 50 mmol/l Tris-HCI (pH 7.4), 1 % Triton X-100, 0.25 % (wt/vol) sodium deoxycholate, 150 mmol/l NaCl, 1 mmol/l EGTA, 0.1 % SDS and protease inhibitors. Protein concentration was measured with the bicinchoninic acid (BCA) method using a Protein Assay Reagent kit (Pierce). Cell lysates (5 or 10 μg) were resolved via SDS-PAGE and transferred to PVDF membranes. Membranes were blocked with 5 % nonfat dry milk (Carnation), and then incubated for 1–2 h with anti-RGC-32 polyclonal antibody (Santa Cruz Biotechnology), NGAL, α-smooth muscle actin (α-SMA), E-cadherin primary antibodies (Abcam) or fibronectin (FN), and tubulin polyclonal antibodies (Sigma) in blocking buffer. Horseradish peroxidase-conjugated secondary antibody was added for the incubation. The blots were visualized via enhanced chemiluminescence (Pierce) and analyzed using a Fuji Photo Film Co. imaging system.

### Immunofluorescent staining

NRK-52E cells were fixed in 4 % paraformaldehyde in phosphate-buffered saline (PBS) at room temperature for 30 min, followed by exposure to methanol (−20 °C) for 10 min. Cells were then blocked in PBS with 1 % bovine serum albumin, 4 % normal serum and 0.4 % TritonX100 for 30 min. Then the cells were incubated with primary antibodies for 2 h at room temperature. Fluorescent dye-conjugated anti-rabbit secondary antibodies were used to detect staining. The cell nucleus was stained with 4′,6-diamidino-2-phenylindole (DAPI). Cell morphology was visualized using fluorescence microscopy.

### Fluorescence-activated cell sorting analysis for cell cycle distribution

NRK-52E cells were prepared for propidium iodide (Sigma) staining according to standard protocols. DNA content was determined with a FACSCaliber (Becton Dickinson FACScan) Analyzer. Cells in G0/G1 or G2/M phase were isolated through ultraviolet-MoFlo sorting (DakoCytomation High Speed MoFlo Sorter).

### Statistical analyses

Data were expressed as means ± SE. All experiments were repeated independently three or four times. Analysis of variance was used to assess the differences among multiple groups. Student’s *t*-test was used to assess the differences between pairwise groups. *p* < 0.05 was considered statistically significant.

## Results

### RGC-32 expression in the injured NRK-52E cells induced by TNF-α

**Fig. 1:**
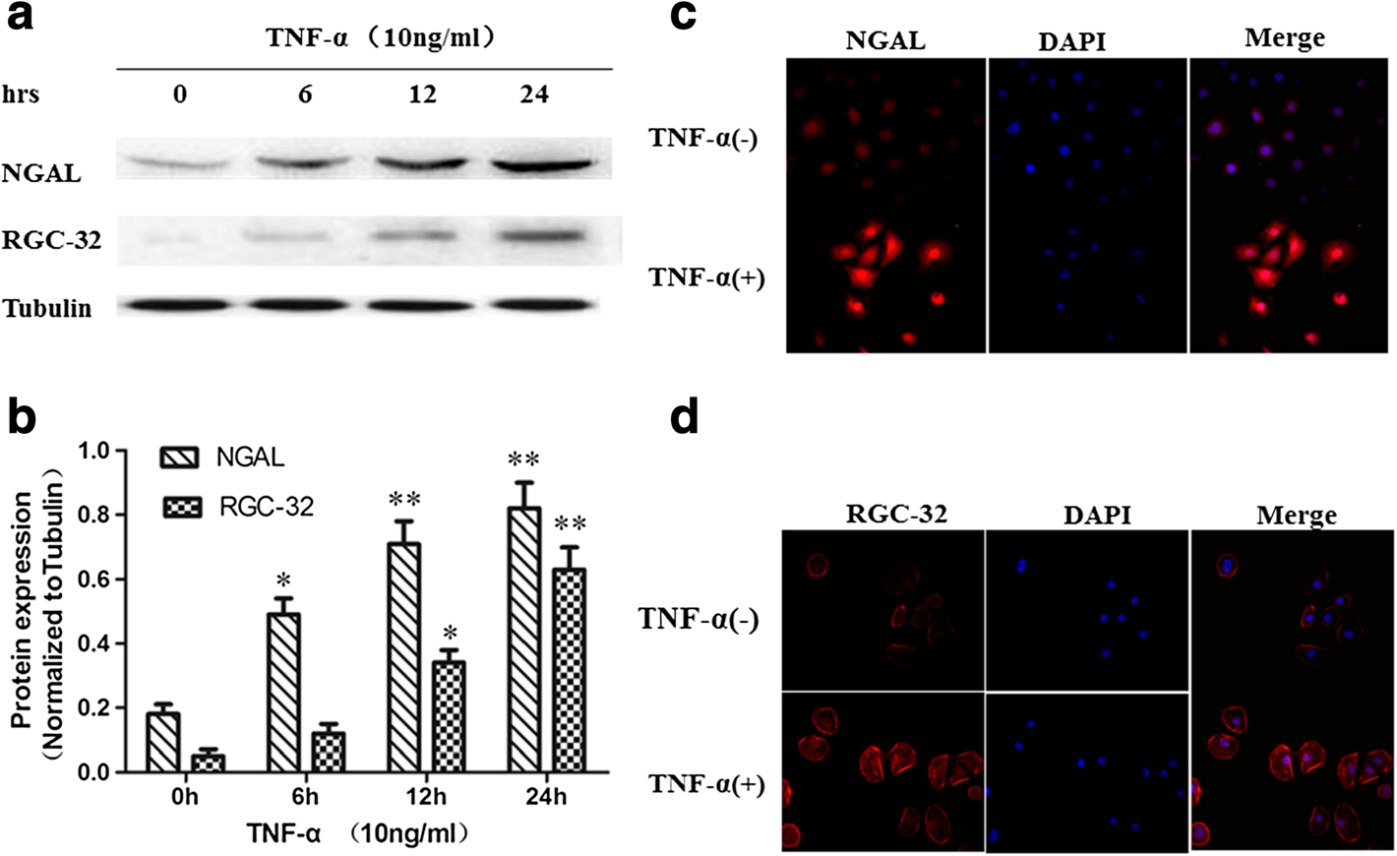
NGAL and RGC-32 expression in NRK-52E cells treated with TNF-α. **a** – NRK-52E cells were treated with TNF-α (10 ng/ml) and the protein expression of NGAL, RGC-32 and tubulin was detected via western blotting. **b** – NGAL and RGC-32 expression were normalized to tubulin (*n* = 5, *p < 0.05, **p < 0.01). **c** – NGAL localization was analyzed via immunofluorescence staining. NRK-52E cells were treated with TNF-α (10 ng/ml) or untreated for 24 h, and then stained with anti-NGAL primary antibody, followed by incubation with Cy3-conjugated secondary antibody. **d** – RGC-32 localization was evaluated via immunofluorescence staining. NRK-52E cells were treated with TNF-α (10 ng/ml) or untreated for 24 h, and then stained with anti-RGC-32 primary antibody, followed by incubation with Cy3-conjugated secondary antibody

To determine whether TNF-α could cause acute cell injury, NRK-52E cells were cultured with TNF-α (10 ng/ml), and the expression level of NGAL was determined via western blotting and immunofluorescent staining (Fig. 1). The NGAL protein level significantly increased in a time-dependent manner after 6–24 h of incubation (Fig. 1a and b). Immunofluorescent staining also showed that increased NGAL levels in NRK-52E cells treated with TNF-α for 24 h (Fig. 1c). These results indicated that the treatment of TNF-α could cause cell injury.

To determine whether RGC-32 expression and cellular localization change in NRK-52E cells after treatment with TNF-α, western blotting and immunofluorescence analysis were performed. There was little expression of RGC-32 in NRK-52E cells that had not undergone TNF-α treatment. Western blotting showed that RGC-32 protein expression significantly increased when NRK-52E cells were treated with TNF-α for 6–24 h (Fig. 1b). RGC-32 was restricted to the cell membrane, and there was also weak expression in the cytoplasm and nucleus. Immunofluorescent staining showed similar results for NRK-52E cells treated with TNF-α for 24 h: significantly increased expression of RGC-32 (Fig. 1d).

### Cell cycle distribution

**Fig. 2:**
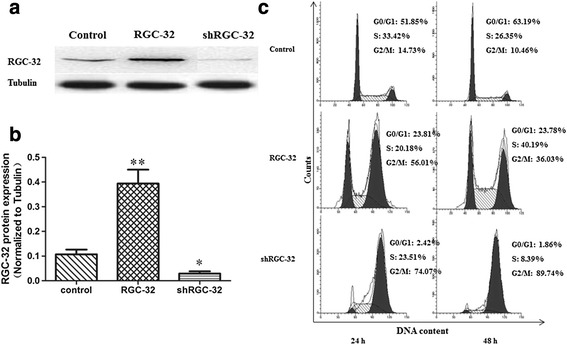
The expression of RGC-32 has an impact on cell cycle distribution. **a** – NRK-52E cells overexpressing RGC-32 or with silenced RGC-32 were constructed via transient transfection of RGC-32 expression plasmid and RGC-32 siRNA plasmid. Western blotting was performed to detect RGC-32 and tubulin protein expression. **b** – RGC-32 protein expression was normalized to tubulin (*n* = 5, *p < 0.05, **p < 0.01). **c** – The cell cycle distribution of NRK-52E cells was analyzed via flow cytometry. Five blank plasmids constitute the control plasmid

To further investigate the role of RGC-32 in cell cycle distribution, we constructed NRK-52E cells with overexpressed or silenced RGC-32 via transient transfection (Fig. 2a and b), and the cell cycle distribution was determined via fluorescence-activated cell sorting analysis (Fig. 2c). Compared with the control cells, the cells overexpressing RGC-32 showed an obvious increase in the number of cells in G2/M phase, while the number of cells in G0/G1 phase decreased markedly. In the cells with silenced RGC-32, the number of cells in G2/M phase increased while the number in other phases decreased dramatically, indicating that RGC-32 silencing probably held cells at G2/M.

### α-SMA, FN and E-cadherin expression in NRK-52E cells with silenced RGC-32

**Fig. 3:**
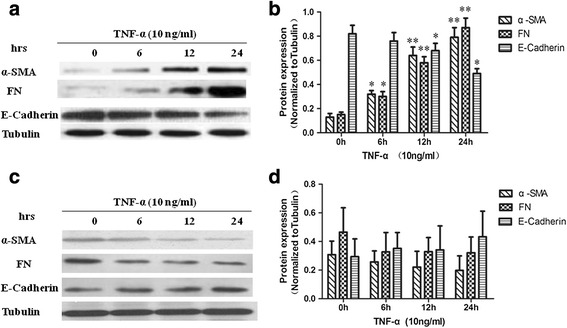
α-SMA, FN and E-cadherin protein expression in NRK-52E cells overexpressing RGC-32 or with silenced RGC-32. **a** – NRK-52E cells with silenced RGC-32 were treated with TNF-α (10 ng/ml). Then α-SMA, FN, E-cadherin and tubulin protein expression were detected via western blotting. **b** – α-SMA, FN and E-cadherin protein expression were normalized to tubulin (*n* = 5, *p < 0.05, **p < 0.01). **c** – NRK-52E cells overexpressing RGC-32 were treated with TNF-α (10 ng/ml). Then α-SMA, FN, E-cadherin and tubulin protein expression were detected via western blotting. **d** – α-SMA, FN and E-cadherin protein expressions were normalized to tubulin (*n* = 5)

The cells with silenced RGC-32 were treated with TNF-α (10 ng/ml) for 0, 6, 12 and 24 h. The expressions of α-SMA, FN, E-cadherin and tubulin were examined via western blotting (Fig. 3a). The results showed that the expression of α-SMA and FN increased in a time-dependent manner, while the expression of E-cadherin decreased gradually with time (Fig. 3b).

The cells overexpressing RGC-32 were treated with TNF-α (10 ng/ml) for 0, 6, 12 and 24 h. Western blotting revealed that TNF-α treatment did not make any difference in the expression levels of α-SMA, FN, E-cadherin or tubulin in those cells (Fig. 3c and d).

## Discussion

The human and mouse RGC-32 gene was first cloned and analyzed by Badea et al. [18]. The human RGC-32 gene, located on chromosome 13, encodes a 137-amino acid protein. Subsequent research verified that the expression of RGC-32 is regulated by activated complement and C5b-9 [12], steroid hormones [19], growth factors [20] and TGF-β1 [16]. RGC-32, an important checkpoint for the cell cycle [18, 21], plays a critical role in cell proliferation and the inflammatory reaction induced by C5b-9 [12]. It also involved in directing the differentiation process [16, 22, 23]. In this study, RGC-32 was activated by TGF-β1 and its expression was increased with TNF-α treatment time, indicating that RGC-32 was involved in the TNF-α-induced acute injury of NRK-52E cells.

RGC-32 plays an indispensable role in renal tubular epithelial cell injury. Niu et al. demonstrated that RGC-32 is expressed in epithelial tubular cells, but not in the glomerulus, renal interstitium or vessels of normal renal tissue. In addition, the expression of RGC-32 is correlated with the degree of pathological lesions of IgA nephropathy [24]. TGF-β plays a valuable role during the entire process, including early injury, repair and tubulointerstitial fibrosis [25–27]. RGC-32 is a downstream target of TGF-β and is activated by Smad signaling. It is also a novel activator of epithelial–mesenchymal transition (EMT) in renal tubular cells: it activates transcription of some EMT-related genes [7]. Both in vitro and in vivo studies found that RGC-32 is a critical regulator of the G2/M checkpoint as well as the activation of TGF-β [7, 8, 13, 24, 28, 29].

In our study, compared with the controls, there was an obvious increase in the number of RGC-32-overexpressing cells in G2/M phase, while the number of cells in G0/G1 phase decreased markedly. These results further suggest that RGC-32 may promote cell proliferation by promoting S-phase entry and G2/M phase cycling. RGC-32 silencing stalled the cell cycle at the G2/M phase [27]. Our study also proved a dramatically increased number of cells in G2/M phase after RGC-32 silencing.

As a critical G2/M checkpoint regulator, RGC-32 participates in cell cycle regulation. Several lines of evidences support this. Previous studies showed that RGC-32 is a novel p53-inducible gene and that it is located at the centrosomes during mitosis and involved in G2/M arrest through its formation of a protein complex with polo-like kinase 1 and phosphorylation in vitro [9–11]. Recently, Vlaicu et al. suggested that RGC-32 plays an important role in regulating the G2/M checkpoint in M-phase by regulating chromatin assembly [10]. Schlick et al. proved that RGC-32 is able to promote the activation of B-cells by regulating the activity of the Cdk1 complex, and thus exerts control on the G2/M checkpoint [30].

In addition, overexpression of RGC-32 in human aortic smooth muscle cells (SMCs) increased the incorporation of BrdU and the number of cells progressing into G2/M phase [20]. RGC-32 complexes with CDC2/cyclin B1 and increases its kinase activity, which is dependent on phosphorylation of RGC-32 at threonine-91 by CDC2, indicating that RGC-32 may serve as a substrate and regulator of CDC2 [20]. RGC-32 is translocated from the cytoplasm to the nucleus to bind and activate key mitotic kinases during mitosis [8], and is connected to the centrosomes [9].

Similar results of RGC-32 expression in the cell membrane, cytoplasm and nucleus were obtained in this study. This indicates the important role of RGC-32 in cell cycle during renal tubular epithelial cell repair. These findings suggest that RGC-32 dynamically regulates the G2/M checkpoint not only by directly regulating activation of the cyclin B1–Cdk 1 complex, but also by indirectly regulating p53 and PLK1.

Cell cycle arrest at G2/M due to RGC-32 silencing in epithelial cells mediates kidney fibrosis after injury [27]. The expression levels of extracellular matrix and fibrosis factors in NRK-52E cells with silenced RGC-32 were further explored. The findings showed that the expression levels of α-SMA and FN increased in a time-dependent manner after TNF-α treatment, while the E-cadherin level decreased gradually. However, TNF-α had no effects on the expression of these proteins in NRK-52E cells overexpressing RGC-32.

Tubular cell EMT is important for the development of renal fibrosis. The complete transition is thought to occur rarely, but partial EMT is required in tubular epithelial injury repair, while the production of fibrotic cytokines by damaged tubular cells is increased [14]. Our study provided additional evidence to support this theory. In the cells with silenced RGC-32 that were treated with TNF-α, the increased expression of α-SMA, which is a marker of myofibroblast formation, indicated the fibrosis of the renal interstitium. FN expression may be associated with tubulointerstitial fibrosis [31]. E-cadherin is a calcium-dependent cell–cell adhesion glycoprotein, and its decreased expression implies a reduction in cell adhesive ability. These results indicated that TNF-α treatment caused cell fibrosis and reduced cell adhesive ability in cells with silenced RGC-32 arrested in G2/M phase.

## Conclusion

In summary, our findings indicate that RGC-32 may play an indispensable role in renal tubular epithelial cell injury and repair by regulating the G2/M checkpoint in the cell cycle. The exact molecular mechanisms remain to be clarified.

## Abbreviations

DAPI: 4′,6-diamidino-2-phenylindole
DMEM: Dulbecco’s modified Eagle’s medium
EMT: Epithelial-mesenchymal transition
FN: Fibronectin
NGAL: Neutrophil gelatinase-associated lipocalin
PBS: Phosphate-buffered saline
RGC-32: Response gene to complement 32
RGCC: Regulator of cell cycle
SMCs: Smooth muscle cells
TNF-α: Tumor necrosis factor-alpha
α-SMA: Alpha-smooth muscle actin
